# A protocol for loading Calcein-AM into extracellular vesicles from mammalian cells for clear visualization with a fluorescence microscope coupled to a deconvolution system

**DOI:** 10.1371/journal.pone.0317689

**Published:** 2025-01-24

**Authors:** María-Angélica Calderón-Peláez, Jaime E. Castellanos, Myriam L. Velandia-Romero

**Affiliations:** Virology Group, Vice-chancellor of Research, Universidad El Bosque, Bogotá, Colombia; Mass Eye Infirmary, Harvard Medical School / Northeastern University, UNITED STATES OF AMERICA

## Abstract

Extracellular vesicles (EVs) are membrane-bound structures produced and released into the extracellular space by all types of cells. Due to their characteristics, EVs play crucial roles in cellular communication and signaling, holding an immense potential as biomarkers and molecular transporters. Various methods have been developed to label and characterize EVs, however, visualizing EVs remains a process that requires highly specialized and expensive equipment, which is not always available in all the laboratories. In this study, we adapted a protocol originally designed for EVs analysis by flow cytometry using Calcein-AM, and convert it into a useful and effective tool for visualizing EVs by epifluorescence microscopy coupled with a deconvolution system. This approach can be very useful for basic EVs analyses, enabling researchers to verify their distribution and internalization across cells. Such insights can guide decisions on whether to advance to more detailed analysis using confocal microscopy or to perform additional assays.

## Introduction

Over the past few years, EVs have attracted significant attention both biologically, as potential biomarkers, and biotechnologically due to their enormous therapeutic potential [1]. These versatile membrane-bound structures are: i) heterogeneous (in size, content, and function), ii) capable of traversing different biological barriers and reaching target cells, and iii) possess low immunogenicity and high immune evasion capacity, making them ideal candidates to act as vehicles for delivering various content types referred to as "cargo" [2].

To date, the development and implementation of techniques for studying EVs have enabled their characterization by size and content -including proteomic, lipids, and nucleic acid analysis- [3]. However, tracking the EVs internalization and their distribution within cells, both *in vitro* and *in vivo* remains challenging [4].

Several approaches have been proposed for visualizing EVs, including labeling the parental cells [5], incorporating bioluminescent or fluorescent proteins into the EVs structure through genetic engineering, direct labeling with antibodies, or using lipophilic fluorescent markers [4]. For details about the different types of markers used for labeling EVs, we recommend the reviews of Bao et al [6] and Boudna et al [7].

Most of the proposed strategies are versatile, enabling EVs detection by flow cytometry (for quantification and size-based separation) and precise visualization within cells by confocal microscopy. However, they also have some disadvantages, such as non-specifically labeling of cellular debris [4, 8] or forming aggregates, which are detected by the equipment, introducing "noise" into the results [5]. Additionally, it is unclear whether these markers interfere with the recognition and unloading of contents in the target cell. Furthermore, some of these markers become exclusive reagents for labeling EVs, increasing costs, along with the mandatory use of specialized and high-cost equipment for visualization [9].

Calcein-AM or CA (Calcein-acetoxymethylester) is a frequently used fluorescent marker to evaluate cellular viability [10, 11]. This non-fluorescent compound is membrane-permeable and, upon hydrolysis by intracellular esterase, converts into its anionic fluorescent non-permeable compound that is retained into the cell [12]. Given the lipid bilayer and protein composition of EVs, CA can specifically enter EVs, where it is processed by intraluminal esterase, remaining in its anionic form within intact vesicles. This specificity has enabled the use of this marker in flow cytometry to identify and quantify EVs based on size, as has been previously reported [8, 13, 14].

Reports of EVs loaded with CA visualized by fluorescence microscopy and not by confocal microscopy, are rare. While confocal microscopy has clear advantages, it is not always accessible in all laboratories, and its use often requires thorough technical verification. In contrast, epifluorescence microscopy, when combined with advanced image processing techniques such as deconvolution (a technique that enhances image clarity and resolution by mathematically correcting blurring) [15], offers notable benefits over confocal microscopy, presenting a cost-effective accessible alternative for cell imaging.

Therefore, if the interest lies in determining whether the evaluated EVs were internalized in target cells and the time it takes to do so, we propose a simple and cost-effective protocol for visualizing CA-loaded EVs, achieved by using an epifluorescence microscope coupled with a deconvolution system, which allows for 3D reconstruction of images. Such a setup can serve as a useful tool for verifying EVs distribution and internalization within cells. It can also facilitate the decision-making process regarding whether to scale up to confocal microscopy and perform additional assays.

## Materials and methods

### Ethics statement

The procedures described in this study were approved by the Ethics Committee of the Universidad El Bosque (Bogotá, Colombia; registration No. 013–2019) and the Ethics Committee of the National Institute of Health of Colombia (registration No. 11–2019), in compliance with international and Colombian standards for animal care and use.

### Animals and welfare considerations

1. **Housing and husbandry:** The animals were housed in the animal facility of Universidad Nacional de Colombia, in a private room meeting all the necessary requirements for proper animal care. Adult male and female mice, along with their pups, were kept in appropriately sized ventilated cages (500 cm^2^ of floor space, model NexGen500, Allentown), covered with sterile wood chip (Aspen Chip and Lab Grande Aspen, NEPCO). The top of the cage held an external plastic 250 ml water bottle and a Waltman filter that allowed clean air exchange and protected the food (placed in a half pocket wire bar lid) with all the nutritional requirements for mice’s survival and reproduction. The cage had an enrichment (60 mm x 78 mm) for mice entertainment. All home cages were properly labeled, and the birth dates and the number of offspring at each birth were recorded. During the experimental period, the animals moved to clean cages with new food and water once a week.
2. **Animal physical condition monitoring**: Every day during experimentation period, we observed and recorded the typical behaviors of the animals, including maintenance, exploration, affiliate interactions, and sexual and maternal behaviors. Any abnormal behavior was promptly reported to the veterinarian.
3. **Steps to minimize pain and distress:** During the experimental period, two sick animals were observed before the endpoint. Sick animals, were euthanized by overdose of anesthetic followed by cervical dislocation in a private room at the animal facility. The bodies were stored in the cold environment until incineration.
4. **Protocol for the early euthanasia/humane endpoints for animals who became severely ill/moribund during the experiments**: If the animals exhibited signs such as weight loss, poor appetite, impaired movement, visible tumors, or abnormal behavior for more than two days, the veterinarian was informed, and animals were set for immediate euthanasia, by an overdose of anesthetic, followed by cervical dislocation to ensure the animal’s quick and humane death while minimizing suffering. However, this protocol was not required in our study.
5. **Euthanasia method for pups utilized in this research:** Postnatal pups of 1 day-old, used for the cultures, were euthanized with an overdose of anesthetic (ketamine and xylazine).

### EVs isolation

Primary cultures of astrocytes and microvascular brain endothelial cells (MBECs) were prepared from mice following protocols previously described by Velandia et al. [16]. After confluency, supernatants from primary astrocyte cultures were collected and centrifuged at 400 ×g for 15 minutes and filtered through 0.22 μm membranes (Merck Millipore). Subsequently, the supernatants were centrifuged at 4,000 ×g to remove larger EVs and concentrated using Amicon Ultra-15 MWCO 100K filters (Merck Millipore) at 4,000 ×g. Finally, ultracentrifugation (UC) was performed at 100,000 ×g for 60 minutes at 4°C using an Optima MAX-TL centrifuge (TLS-55 rotor, k Factor 100.2, Beckman Coulter). The resulting EV pellets were resuspended in 100 μL of filtered, pyrogen-free 1X PBS [17].

### CA loading of EVs and microscopy visualization

The protocol described in this peer-reviewed article is included for printing as S1 File with this article. DOI: dx.doi.org/10.17504/protocols.io.q26g71qz9gwz/v1.

### Other techniques used to detect the EVs

#### Nanoparticle Tracking Analysis (NTA)

This analysis was performed using the Nanosight NS300 and NTA 3.4.4 software (Malvern), following the manufacturer’s instructions. Briefly, 50 μL of CA-loaded EVs were diluted in filtered PBS 1X to a final volume of 1 mL. After assembling the system, the low-volume cell was washed with apyrogenic water, followed by filtered PBS 1X, which was read as a blank to verify the absence of particles in this fluid. Then, the EVs dilution was passed through the system, and a first measurement of all EVs present (loaded or not with CA) was performed. This involved a 5-capture analysis of 60 seconds each, selecting the "Scatter" option in the Focus menu. Once these data were obtained, the same capture parameters were used to re-analyze the same EVs suspension, this time evaluating only those EVs that presented green fluorescence. For this, the camera level was set to maximum, the appropriate fluorescence filter (565 nm long pass) was used, and the "Fluorescence" option was selected in the Focus menu. After obtaining the readings, following the manufacturer’s instructions, both graphs were overlapped to demonstrate the EVs loaded and not loaded with CA over time.

#### Flow cytometry

100 μL of CA-loaded EVs were evaluated by flow cytometry following the protocol by Gray et al., [8]. For this, 100,000 EVs events were analyzed using a Dx Flex cytometer (Beckman Coulter) and CytExpert 2.3 software for the CytoFLEX platform, which has an enhanced sensibility for size resolution, measuring microparticles from 40 nm to 1 μm. The sizes of the CA-loaded EVs were obtained by comparing them to fluorescent reference beads, "GigaMix," (BioCytex), which are a mixture of fluorescent Megamix-Plus SSC (7803) and Megamix Plus FSC (7802) beads (BioCytex a Stago group company, Marseille, France) and were used following the manufacturer’s instructions. Briefly, the Gigamix beads enabled precise selection of the region where EVs would appear in the scatter plot, allowing us to create a reference dot plot that helped differentiate particles for further analysis. Then, gates were drawn around each bead population (S1A and S1B Fig); using this reference, we created a CA-reference dot plot and processed non-loaded controls or the CA-loaded EVs (S1C Fig).

#### Cell Immunofluorescence (IF)

After exposing cells to CA-loaded EVs (S1 File), cells were fixed with 4% paraformaldehyde (PFA), PBS washed, permeabilized with Triton X-100 (0.3%), and blocked with 10% goat serum. Primary antibodies against ZO-1 (Zonula Occludens) (40–2200, Invitrogen-Thermo Fisher Scientific) or GFAP (glial fibrillary acidic protein) (Z0334, Dako) were used following the protocol by Velandia et al., [16]. Then, cells were incubated with the respective secondary antibody conjugated to Alexa 594 (A-11012, Invitrogen-Thermo Fisher Scientific), and nuclei were stained with DAPI (Merck, 10236276001). ProLong Gold Antifade Reagent #9071 (Cell Signaling) was used as fluorescence mounting medium. All images were captured using a Zeiss Axioimager M2 microscope, coupled to the Colibri 7 fluorescence system and the Apotome 2 deconvolution system (Zeiss). Image analysis was performed using Zen 2.6 (blue edition) software.

### Expected results and discussion

**Fig 1:**
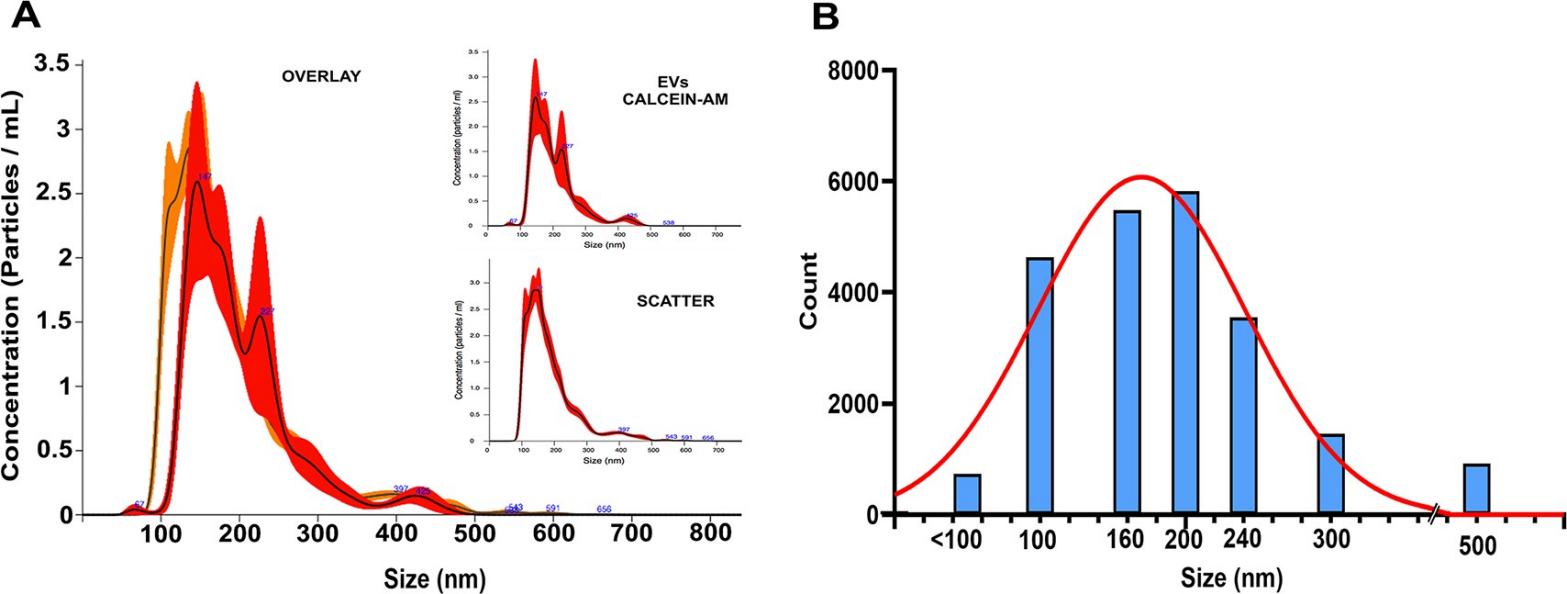
Analysis of EVs by other techniques. A) NTA assay. The EVs dilution was analyzed in Scatter mode to analyze all EVs in the sample and then in Fluorescence mode to show only the EVs loaded with CA. Observing the overlay of the two readings, most vesicles in the sample were loaded with the marker. B) Size profile of CA-loaded EVs evaluated by flow cytometry.

The NTA assays showed that the murine astrocytes produced a total of EVs ranging in size from 90 to 300 nm, with a mean size of 185.5 nm and a mode of 148.7 nm (Fig 1A). The concentration was found to be 3.48 x 10^8 particles/mL. The same sample was also analyzed specifically for EVs loaded with CA. The results showed that these EVs had sizes between 100 and 300 nm, with a mean of 200.5 nm, a mode of 146.1 nm (Fig 1A), and a concentration of 2.82 x 10^8 particles/mL. These results indicate that more than 80% of EVs present in the suspension were labeled with CA.

Complementarily, flow cytometry also quantified the CA-loaded EVs, which were sorted by size (Fig 1B), according to the Gigamix reference beads, with seven different sizes: 100, 160, 200, 240, 300, 500, and 900 nm, recommended for daily standardization of microparticle measurement on the CytoFLEX (Beckman Coulter). The results indicated that the majority of labeled EVs fell within the 100 and 300 nm range, consistent with the NTA findings. Specifically, there were 4630 events at 100 nm in size, 5439 events at 160 nm, and 5824 events at 200 nm (Fig 1B).

Next, 50.000 murine cerebral microvascular endothelial cells (MBEC) or 30.000 astrocytes from 1-day-old Balb/C mice, obtained as published by Velandia et al, [16] and seeded 24 hours prior were inoculated with 3 million CA-loaded EVs (S1 File) to assess their cellular distribution at 30-, 90-, and 180-minutes post-exposure. At the end of these times, cells were fixed and processed for IF.

**Fig 2:**
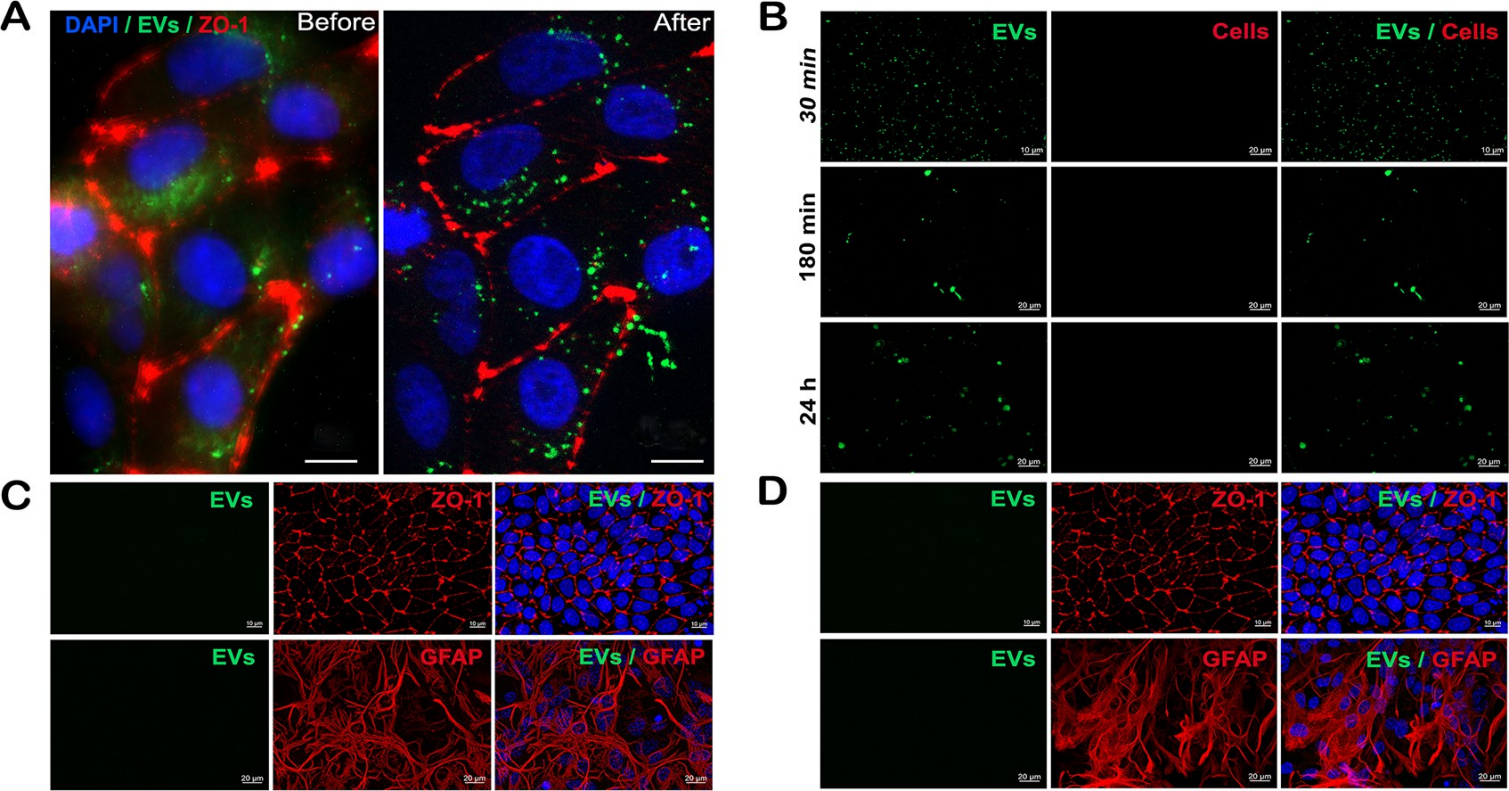
Cells with and without deconvolution and the controls used for EVs visualization. A) MBEC cells before and after the deconvolution process. B) CA-loaded EVs seeded on pre-coated slides without cells, detected after 30, 180 min and 24 h. C) cells exposed to non-loaded EVs and D) cells without EVs exposure.

As mentioned, deconvolution removes blurring and distortions from images, allowing for the evaluation of details that are otherwise undetectable in standard epifluorescence imaging. Fig 2A presents a comparison of a MBEC image inoculated with CA-loaded EVs before and after deconvolution. Prior to deconvolution, it is difficult to differentiate EVs within the cells, as the cellular interior appears blurry, and even the cell margins exhibit distortions. In contrast, after deconvolution, both the EVs and cell margins are more clearly defined, making it possible to distinguish the EVs location (Fig 2A). As controls all experiments were processed along with i) CA loaded EVs placed on Poly-L lysine pre-coated slides without cells (Fig 2B), ii) non-loaded EVs seeded on cells (Fig 2C) or C) cells non-exposed to EVs (Fig 2D).

**Fig 3:**
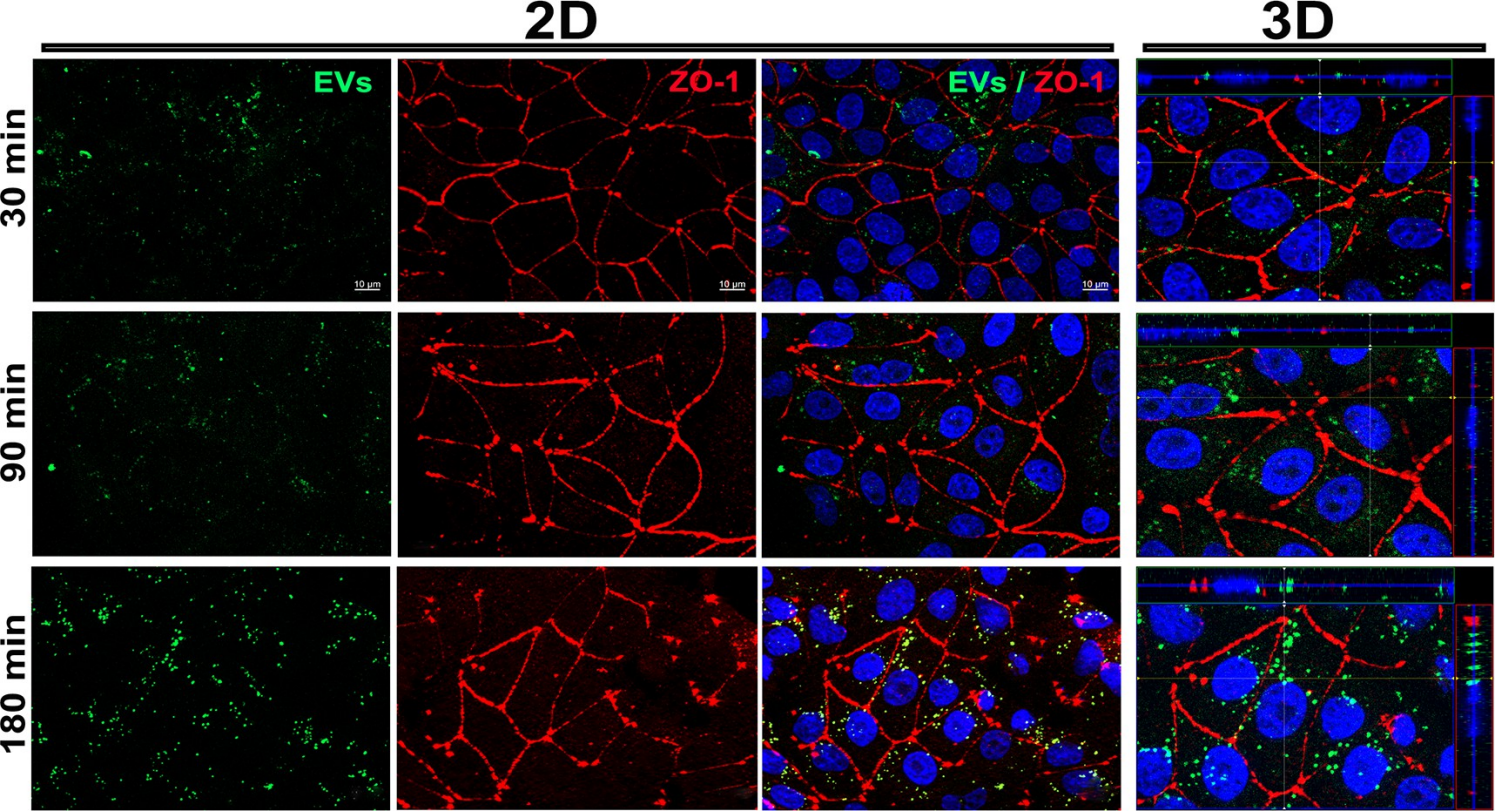
Early post-exposure EVs distribution in MBEC cells. EVs derived from astrocytes were loaded with CA and subsequently incubated with MBEC cells. At different times (30, 90, and 180 minutes), the internalization of EVs (green) into cells was monitored, with cell boundaries delineated by labeling the endothelial protein ZO-1 (red) and nuclei (blue). 3D analysis facilitated for the observation of EVs localization within cells.

The deconvolution processing revealed that between 30 and 90 minutes, the EVs were dispersed across the MBEC cells, with numerous punctiform particles of varying sizes observed (Fig 3). The 3D analysis indicated that at 30 minutes, the majority of EVs were located on the cell surface, whereas by 90 minutes, EVs were observed both inside the cell and on the cell surface (Fig 3). At 180 minutes, a greater aggregation of EVs was found inside the cell, and in some cells, they were observed accumulated in the perinuclear region. This was confirmed by 3D analysis, where a greater number of EVs or aggregates were observed inside the cells (Fig 3).

In general, the results showed a time-dependent increment in the amount of EVs observed in the cell monolayer. This variation can be influenced by several factors, including the density of the culture medium and the physical properties of the EVs themselves [18]. Culture medium density significantly influences the sedimentation and diffusion rates of EVs; a denser medium may initially hinder the rapid movement of EVs towards the cells, affecting their uptake kinetics [19]. As time progresses, EVs can gradually overcome these barriers through mechanisms such as endocytosis or membrane fusion, leading to increased accumulation within the cells. Additionally, the weight and size of the EVs play a role in their internalization; larger or heavier EVs may be internalized more slowly due to physical constraints or limited accessibility to cellular uptake pathways [19, 20]. Consequently, the settling effect, combined with the cells’ own EVs uptake dynamics, could contribute to the greater fluorescence observed at later time points.

**Fig 4:**
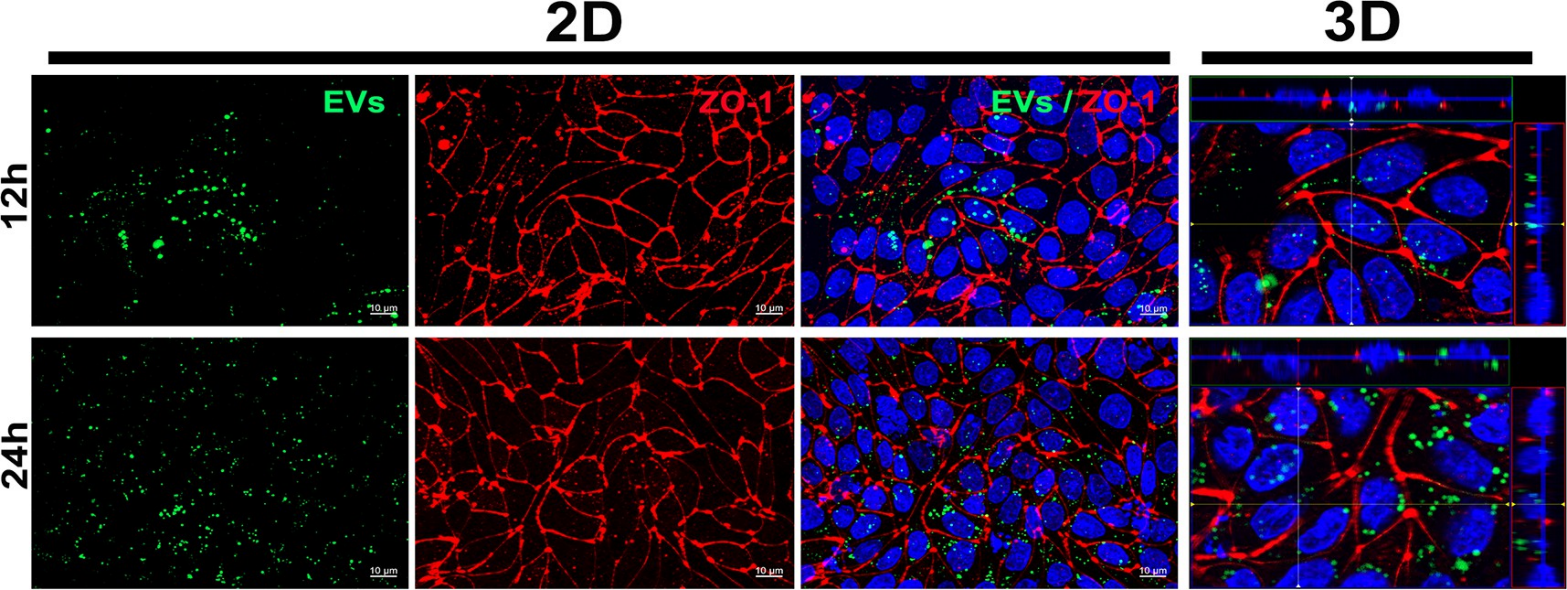
Late post-exposure EVs distribution. CA-loaded EVs (green) were incubated for 12 h or 24 h with MBEC cells marked with the protein ZO-1 (red). According to the 3D analysis, in these times all EVs were located within the cells.

Based on the results, we sought to determine if CA-loaded EVs could still be observed at later post-exposure times. To investigate this, we evaluated 12 and 24 hours post exposure. Surprisingly, during these periods we observed numerous fluorescent aggregates (Fig 4), exclusively within the cell. It is possible that at these time points, EVs or the released CA are being recognized and processed within endosomes, lysosomes, or other cellular compartments. Therefore, we cannot dismiss the possibility that these aggregates are EVs or fragments of them encapsulated within other organelles.

**Fig 5:**
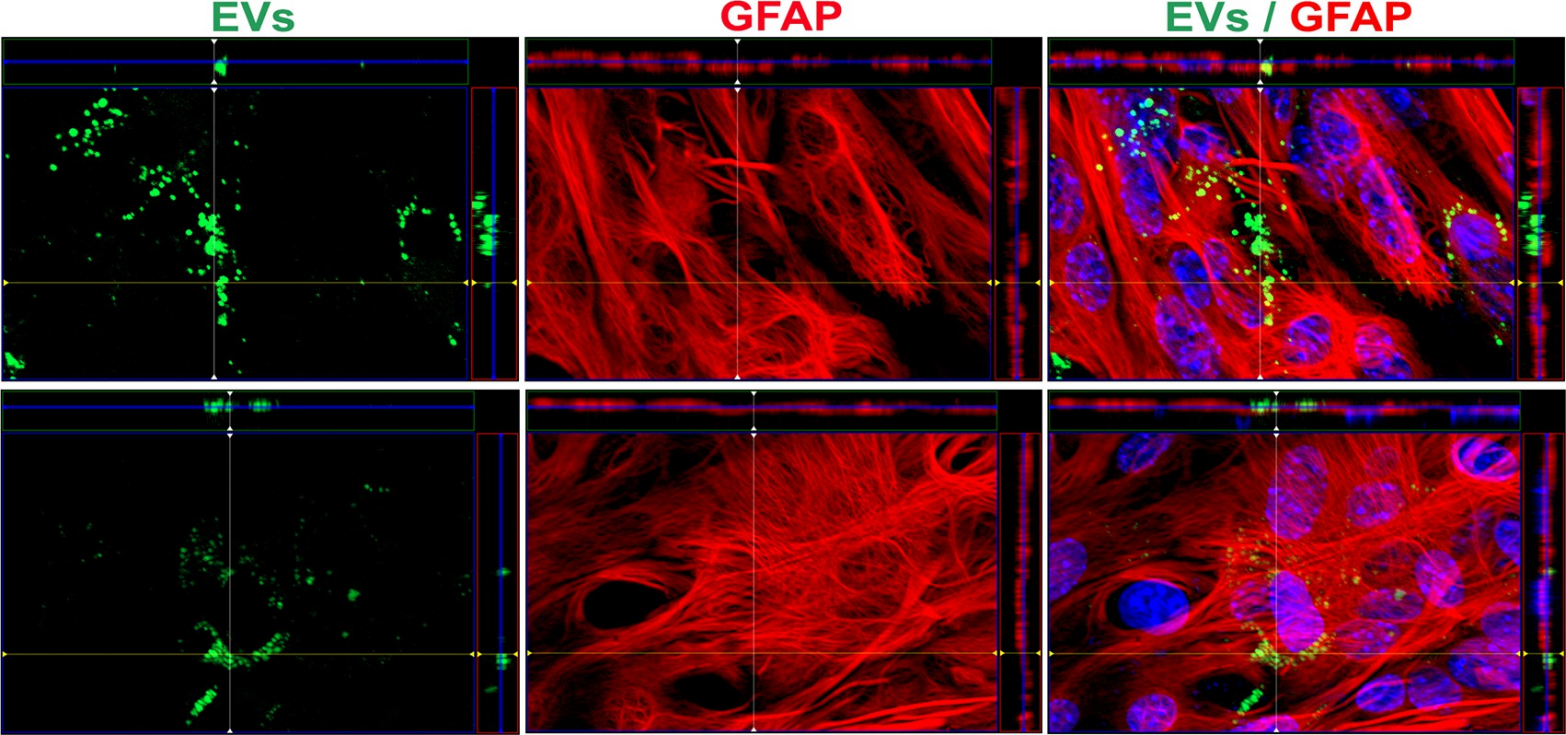
EV distribution in astrocytes. EVs from independent astrocyte cultures were loaded with CA and then incubated with an astrocyte monolayer. Within 90 minutes, the majority of EVs (green) were found inside the cells. Cell extensions were stained with GFAP (red) and nuclei (blue).

Finally, we assessed whether the pattern of distribution and persistence of fluorescent EVs remained consistent when incubated in their originating cells. For this, during 90 minutes, independent astrocyte cultures were exposed to EVs (previously loaded with CA) produced by the same cell type. We found that at this time, like what was observed in the MBEC cells, a significant portion of the EVs were internalized, with some of them predominantly localizing in the perinuclear region (Fig 5).

Historically, research on EVs has predominantly focused on their production and characterization under healthy and diseased conditions. However, relatively few studies have investigated the internalization of labeled EVs, particularly in brain endothelial and glial cells.

In this regard, our proposed method for labeling EVs is both rapid and cost-effective. It takes advantage of CA’s specificity, stability, and sensitivity to label EVs, reducing the likelihood of false labeling. The method is time-efficient, with EVs labeling taking only 30 minutes, allowing for quick initiation of experiments, and it is straightforward and economical, making it suitable for *in vitro* models.

Labeling EVs is crucial for studying their functions, and CA loading has proven to be an inexpensive and minimally disruptive tool for tracking EVs entry into target cells. Jhaveri et al. reported a dose-dependent increase in EV uptake when recipient brain endothelial cells (BECs) were treated with CA-loaded EVs, whereas PKH67-labeled EVs exhibited the opposite trend. These findings emphasize the importance of selecting appropriate dye labeling chemistry when evaluating EVs uptake across cellular models. Unlike CA-labeled EVs, PKH dyes can affect membrane fusion between EVs and recipient cells and alter the particle diameter of labeled EVs [21]. Our findings are consistent with these observations, as we identified numerous CA-labeled EVs within different cell types, demonstrating their stability over time and reinforcing the utility of CA labeling for reliable EVs tracking.

A previous study reported that mouse brain-derived EVs, labeled with DiI, successfully entered glial cells—including astrocytes—via actin-dependent pathways, macropinocytosis and/or phagocytosis. Notably, astrocytes exhibit a lower rate of EVs internalization compared to microglia [22]. This reduced uptake might be explained by astrocytes’ mechanism of slowly endocytosing EVs to mitigate excessive signaling responses, aligning with their crucial regulatory roles in the central nervous system (CNS) [22]. Additionally, another study demonstrated the time-dependent uptake of various concentrations of PKH-labeled EVs by Neuro-2a cells and primary astrocytes *in vitro*, visualized through confocal microscopy. These findings revealed cell-specific variations in EVs uptake over time [23], consistent with the current results presented in this work. These findings may explain the differences observed in our model regarding EVs internalization between MBECs and astrocytes after 90 minutes, where MBECs were found to internalize significantly more EVs within this time frame (Figs 3 and 5).

On the other hand, the origin of EVs appears to be a key factor in determining their affinity for and rate of entry into target cells. A recent study reported that brain endothelial cells (hCMEC/D3 BEC) internalize homotypic EVs—those produced by the same cell type—at a significantly higher rate than heterotypic EVs, such as those produced by macrophages [21]. However, this observation contrasts with our findings, where primary MBECs internalized astrocyte-derived EVs more efficiently within a short period of time. These results suggest that the interaction and rate of EVs entry into target cells are influenced by factors such as cell type, culture type (primary cells versus cell lines), and the homotypic or heterotypic origin of the EVs. Furthermore, the functional and ontogenetic differences between MBECs and astrocytes, their close association within the CNS and their regular interactions may play a role in the observed discrepancies between our findings and those reported by others.

These studies highlight the importance of visualizing EVs within target cells to experimentally confirm their internalization, regardless of the underlying mechanism. This step is essential for advancing research and subsequently evaluating the potential role of EVs contents in influencing the cellular response of the internalized cells [24]. However, confocal microscopy, the most employed method for EVs visualization, is not universally accessible due to its high cost, especially during the early stages of experiment standardization; in fact, beyond its cost-effectiveness, epifluorescence microscopy offers notable advantages over confocal microscopy when paired with advanced image processing techniques like deconvolution.

Deconvolution is a technique that improves images clarity and resolution by mathematically reversing the effects of blurring or distortions introduced by the imaging system resulting in sharper images with enhanced detail [15]. Importantly, this method is applicable even with the simplest optical configuration, reducing costs and streamlining the acquisition process [15]. As consequence, this approach enhances the resolution and contrast of epifluorescence images, allowing for detailed visualization of cellular structures without the need for point-scanning, which can reduce photobleaching and phototoxicity [15, 25]. Furthermore, the use of wide-field illumination in epifluorescence microscopy facilitates faster image acquisition, enabling high-throughput imaging and time-lapse studies with minimal photodamage to live cells [25, 26]. By leveraging deconvolution, epifluorescence microscopy serves as a powerful, accessible alternative to confocal microscopy. Importantly, it can be implemented under low-light conditions, improving image contrast and reducing the likelihood of fluorophore bleaching, which is beneficial in terms of phototoxicity in live cells [15].

Notably, while IF protocols may vary depending on the antibody properties and researcher expertise, the time required for these experiments is consistent with standard durations for confocal microscopy procedures. Image analysis using Zen 2.6 Blue Edition software (S1 File) typically takes approximately 30 minutes, depending on the complexity of the image stack and the user’s proficiency. This protocol provides notable time savings and operational flexibility, making it a practical and efficient approach for initial EVs studies without sacrificing accuracy. Nevertheless, the proposed protocol has some limitations, including restricted penetration, which limits its application for imaging deep body structures [27]. Additionally, we recommend optimizing the protocol by directly labeling EVs after isolation to avoid labeling cellular debris, as suggested by Liu et al. [27].

Conversely, deconvolution also brings some challenges if a deconvolution system (as the one purposed in S1 File) is not available at the time of image acquisition, being necessary to carefully optimize the deconvolution parameters. Recent advancements in tools and software have increasingly aimed to streamline and simplify this process. In general, to standardize parameters across different sample types for high sensitivity in deconvolution methods, it is essential to establish a consistent workflow that optimizes settings based on key sample characteristics such as fluorescence intensity, size, and structural complexity [15]. A set of preliminary tests can help determine ideal exposure times, acquisition settings, and deconvolution parameters tailored to each sample type. Additionally, creating reference templates or parameter presets allows for reproducible results across similar sample types, reducing the need for re-optimization and saving both time and computational costs.

Various authors have proposed workflows to streamline the creation of effective deconvolution protocols. For example, Nguyen et al., described 53 distinct deconvolution methods, benchmarks their performance, and introduced an R package called DeconBenchmark for deconvolution tasks [28]. Additionally, the “DeconvolutionLab” tool, a user-friendly platform for deconvolution microscopy is freely available [15]. Furthermore, Zeiss offers a practical guide that includes recommendations for image acquisition and deconvolution settings, along with tips to deepen understanding and implementation of the technique [29].

## Conclusions

The evaluation of EV uptake is a crucial foundational step in advancing research in this field. While confocal microscopy remains the gold standard due to its superior resolution and numerous advantages, its high cost and time requirements can pose challenges for smaller laboratories, particularly in the early stages of investigation. To overcome these limitations, we propose the use of epifluorescence microscopy combined with deconvolution techniques as a cost-effective and efficient alternative for studying EV internalization in cells. Using this approach, we successfully assessed EV uptake in two distinct cell types at early time points, uncovering significant differences in the amount of internalized EVs between them. Moreover, CA-based EV labeling proved to be highly effective, rapid, and specific. Applying deconvolution to epifluorescence microscopy further enhanced the visualization of EVs, providing an alternative approach to cell imaging comparable to its established use in tissue evaluations. This expands the overall utility and applicability of the technique, broadening its potential for a wide range of biological investigations.

## Supporting information

S1 File Step-by-step protocol for loading EVs with CA and then successfully analyzes them by fluorescence microscopy coupled to a deconvolution system. (PDF)

S1 Fig Scatter plots of flow cytometry results. A) Scatter plot and gating strategy of the Gigamix beads. B) Histogram of the Gigamix beads. C) Scatter plot image of the CA-loaded EVs in the template scatter plot.
(TIF)
