# Supplementary material for: A protocol for loading Calcein-AM into extracellular vesicles from mammalian cells for clear visualization with a fluorescence microscope coupled to a deconvolution system
Source: PLoS One. 2025 Jan 24;20(1):e0317689. doi: 10.1371/journal.pone.0317689 (PMC11761115; doi:10.1371/journal.pone.0317689)
Supplement: S1 File — (PDF) [file pone.0317689.s001.pdf]

Step-by-step protocol for loading Calcein-AM into extracellular vesicles from mammalian cells for its clear visualization with a fluorescence microscope coupled to a deconvolution system

RESERVED DOI:
10.17504/protocols.io.q26g71qz9gwz/v1

María-Angélica Calderón-Peláez[1], Jaime E Castellanos[1], Myriam L. Velandia-Romero[1]

[1]Virology Group, Vicechancellor of research, Universidad El Bosque. Bogotá (Colombia)

María-Angélica Calderón-Peláez
Universidad El Bosque

Protocol Info: María-Angélica Calderón-Peláez, Jaime E Castellanos, Myriam L. Velandia-Romero . Step-by-step protocol for loading Calcein-AM into extracellular vesicles from mammalian cells for its clear visualization with a fluorescence microscope coupled to a deconvolution system. **Protocol exchange https://protocols.io/view/step-by-step-protocol-for-loading-calcein-am-into-dppp5mmn**

Created: October 14, 2024

Last Modified: October 16, 2024

Protocol Integer ID: 110031

Keywords: Calcein-AM, Deconvolution, Extracellular vesicles, Microscopy

Funders Acknowledgement:
Ministerio de Ciencia Tecnología e Innovación de Colombia (Minciencias)
Grant ID: 130884467149
Contrat 431-2020

Abstract

Extracellular vesicles (EVs) are membrane-bound structures produced and released into the extracellular space by all types of cells. Due to their characteristics, EVs play crucial roles in cellular communication and signaling, holding an immense potential as biomarkers and molecular transporters. Various methods have been developed to label and characterize EVs, however, visualizing EVs remains a process that requires highly specialized and expensive equipment, which is not always available in all the laboratories. In this study, we adapted a protocol originally designed for EVs analysis by flow cytometry using Calcein-AM (CA), and convert it into a useful and effective tool for visualizing EVs by epifluorescence microscopy coupled with a deconvolution system. This approach can be very useful for basic EVs analyses, enabling researchers to verify their distribution and internalization across cells. Such insights can guide decisions on whether to advance to more detailed analysis using confocal microscopy or to perform additional assays.

Materials

Materials:

- EVs previously isolated and resuspended in apyrogenic filtrated PBS 1X. In this case EVs were isolated by ultracentifugation.
- Filtrated Apyrogenic PBS 1X.
- Calcein, AM, cell-permeant dye **Thermo Fisher Catalog #**C1430
- Mammalian cells seeded the day before on glass slides previously coated with Poly-L lysine (10 ug/ml). Ensure that the cells reached at least 80% confluence before use. In this case we used astrocytes and microvascular brain endothelial cells (MBEC) from suckling mice.
- Paraformaldehyde (PFA) at 4% in PBS 1X
- Fluorescence mounting medium. We used ProLong Gold Antifade Reagent **Cell Signaling Technology Catalog #**9071 .
- Hoecht 33342 (Thermo Fisher Scientific) or DAPI **Merck MilliporeSigma (Sigma-Aldrich) Catalog #**10236276001 for nuclei staining. These stains will serve as cell references during microscope imaging, ensuring that the nuclei are stained with different fluorescent color than the one chosen for CA.
- Use a specific antibody to detect of a membrane or cytoskeleton protein that will serve as a reference for cell diameter. In our study, we used Glial Fibrillary Acidic Protein **Agilent Technologies Catalog #**DAKO Z0334 for astrocytes, and ZO-1 Polyclonal Antibody **Thermo Fisher Scientific Catalog #**40-2200 for MBEC cells.
- Microscope Axiovision M2 (Zeiss) with the Colibri 7 fluorescence system, coupled to the Apotom 2 deconvolution system.

Other materials:

- Micropipettes of different volumes
- Tips for all micropipettes
- Tubes of 1.5 mL
- Incubator at 37 °C with or without CO_2
- Clean and sterilized slides of 10-18 mm of diameter
- 48 or 24 well plaques
- Laminar flow cabinet to maintain the sterility of the cell culture
- Ice or cooler

Before start

Days before the experiment:

Isolate the EVs from the cell culture of interest and store them at -80 °C until use.

Day 1

1d

1 Seed between 50.000 and 80.000 cells in glass slides previously coated with Poly-L lysine (10 undetermined). The number of cells will depend on the slide area and cell size. Ensure that 80% of the slide is covered with cells. Incubate for 24:00:00 at 37 °C and 5% CO_2.

1d

Day 2

40m

2 Completely thaw and keep the EVs and Calcein-AM (CA) On ice while preparing the reagents. Clean the laminar flow cabinet with alcohol and turn it on ensuring the area sterility.

3 Dilute the CA to a final concentration of [M] 10 micromolar (µM) using filtered PBS 1X, and add it to 50 µL of EVs.

For example: if the CA stock is at a concentration of [M] 1 millimolar (mM)

$$\frac{50\ \mu L * 20\ \mu M}{1000\ \mu M} = 1\ \mu L \text{ de Calcein} + 49\ \mu L \text{ filtered PBS 1X} + 50\ \mu L \text{ of EVs}$$

4 Place the EVs-CA dilution in the incubator at 37 °C during 00:20:00 , protected from light.

20m

5 After the incubation, remove the EVs- CA dilution from the incubator, and take it to the laminar flow cabinet.

6 Increase the dilution with filtered PBS 1X, ensuring that at least 3 million EVs are available for contact with the cells seeded 24 h prior.

- **For example**, for these specific assays we increased the dilution to a final volume of 2 mL in filtered PBS 1X, and distribute this dilution over the seeded cells.

7 To bring the EVs into contact with the previously seeded cells, remove the medium, and wash the cells with filtered PBS 1X.

- Meanwhile, mix the CA-loaded EVs with complete culture medium, special for EVs isolation (which does not contain fetal bovine serum -FBS- or with exosome-free FBS), and homogenize by gentle pipetting.
- Finally, add the EVs-containing medium to the cells and incubate for 30, 90 or 180 min, and further incubate for up to 12 and 24 h, protected from light.

Note

Times can vary depending on every research needs.

8 At the end of the incubation times, remove the medium and wash cells with filtered PBS 1X. Then, add PFA 4% to the cells for 00:20:00 at Room temperature . 20m

9 After the incubation period, remove the PFA and wash the cells with filtered PBS 1X at least 3 times. Subsequently, initiate fluorescence analysis to assess the entry of EVs into cells.

Acquisition and images analysis:

10 The Imager M2 microscope (Zeiss), was equipped with an HRm camera, and it was coupled with the Colibri 7 fluorescence system and the Apotom 2 deconvolution system (Zeiss) to capture the images. Acquire all images using a 63x objective lens.

Use of the Software

11 **The Fig S2 summarize the following steps:**
Capture the images using Zen 2.6 (blue edition) software. In this particular case the Axioimager M2 microscope has a motorized stage, because of this the channels to be used were established from the "Acquisition" menu.

12 Set the channels as follows: blue (nuclei, λ=475), green (CA-loaded EVs, λ=488) and red (reference cellular protein λ=584). If, on the other hand, a non-automated microscope is available, the channel adjustment must be done from the "Locate" menu.

13 Set the nuclei (blue channel) as the reference for focusing the image, initializing their distance at zero (Distance 0 µm) using the Reset/Set Zero menu. Establish focus for the other channels relative to this initial distance.

14 When adjusting the focus of the image, note the specific distance value displayed, which should be set in the "Focus offset" menu. Repeat this process for each channel.

15 To prevent over-exposure of fluorescence and loss of brightness, configure the software to automatically adjust the fluorescence intensity and exposure time for each channel using the "Set Exposure" tool.

16 Finally, establish the Z-axis route (Z-stack), which is necessary for the 3D reconstruction carried out after image acquisition.

- To set this up, activate the Z-stack tab in the "Acquisition" menu, specifying a minimum of 10 stacks.
- The apotome system must be activated to initiate the deconvolution system and acquire the image, which may take a few minutes.

17 After acquiring the image, create the Apotome image.

18 Creating the image opens up various possibilities for image analysis. In this instance, our aim was to observe whether CA-loaded EVs entered the cells they were applied to.

19 To accomplish this, we use the 'Ortho' menu on the image created by the Apotome, which enables us to section the image in X and Y within each Z-stack.

- This helps us determine the vertical position of EVs relative to the cell nuclei and understand their distribution across the cell area.
- Conduct additional analyses using the '3D' menu, which allows for analysis in X, Y, and Z dimensions, enabling image rotation and sectioning at various levels."

20 Finally, generate the final image from the 10 Z-stacks. To do this, navigate to the 'Processing' menu and select the 'Extended Depth of Focus' option. To observe the full depth of the images captured, select the 'Maximum Projection' option. After this step, the results are ready for interpretation.

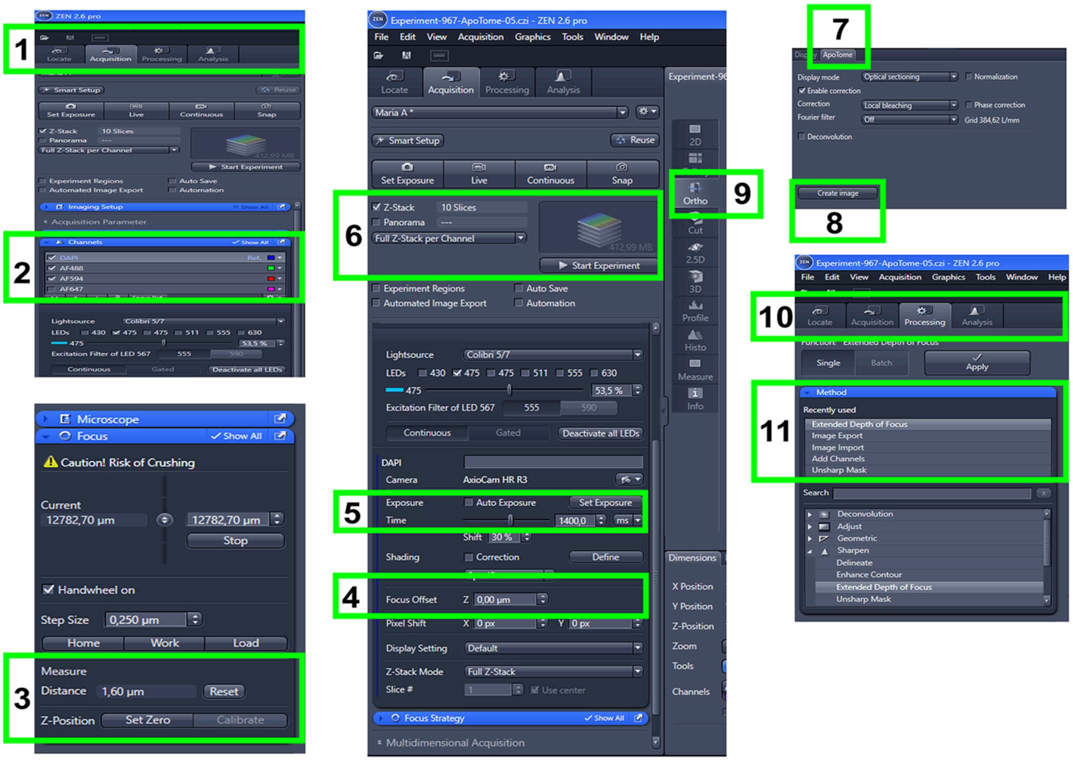

Fig S2. Software workflow for image acquisition.
