# Supplementary figures and images for: A protocol for loading Calcein-AM into extracellular vesicles from mammalian cells for clear visualization with a fluorescence microscope coupled to a deconvolution system
Source: PLoS One. 2025 Jan 24;20(1):e0317689. doi: 10.1371/journal.pone.0317689 (PMC11761115; doi:10.1371/journal.pone.0317689)

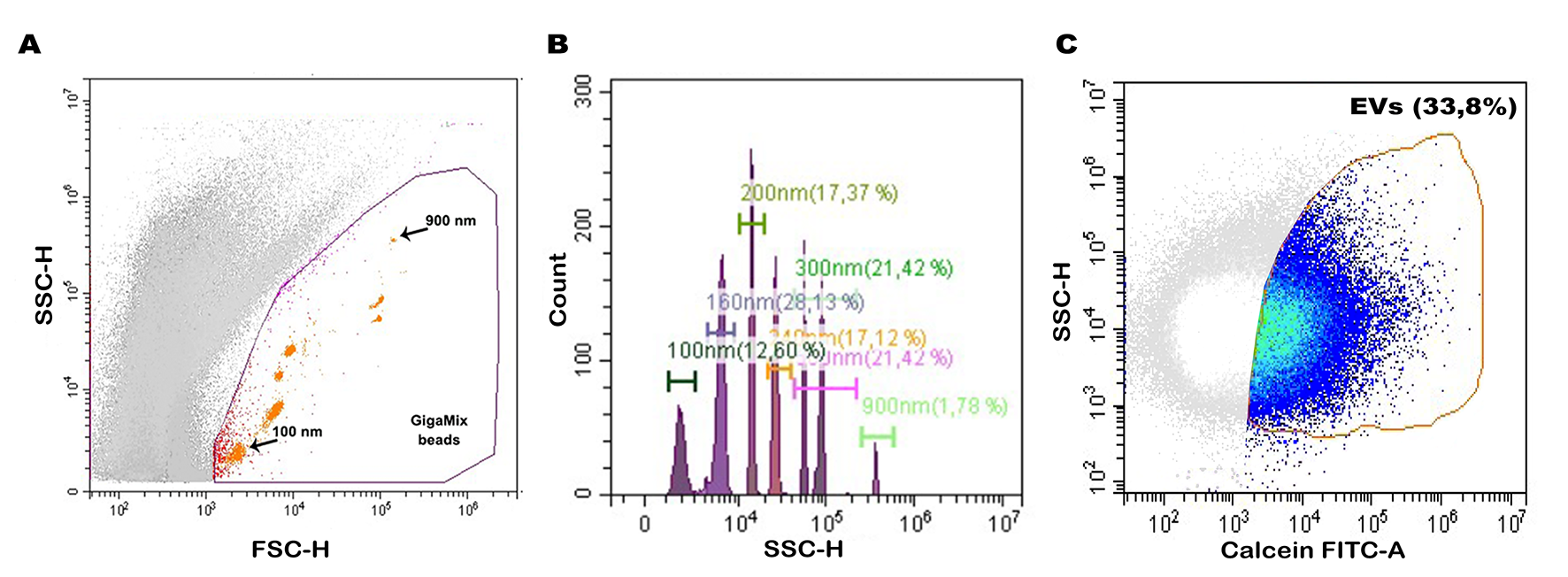

Supplement: S1 Fig — A) Scatter plot and gating strategy of the Gigamix beads. B) Histogram of the Gigamix beads. C) Scatter plot image of the CA-loaded EVs in the template scatter plot. (TIF) [file pone.0317689.s002.tif]
